# A versatile dual-color bacterial reporter system highlights two distinct *Pseudomonas aeruginosa* type 3 secretion system intracellular populations

**DOI:** 10.1128/aem.00490-26

**Published:** 2026-06-03

**Authors:** Christopher J. Corcoran, David G. Glanville, Zachary J. Resko, Erin K. Cassin, Andrew D. Marten, Derrick L. Kamp, Karen L. Visick, Spencer V. Nyholm, Boo Shan Tseng, Abby R. Kroken, Andrew T. Ulijasz

**Affiliations:** 1 Department of Microbiology and Immunology, Loyola University Chicago 2456, Maywood, Illinois, USA; 2 Division of Pulmonary, Allergy and Critical Care, Department of Medicine, University of Alabama at Birmingham 9968, Birmingham, Alabama, USA; 3 School of Life Sciences, University of Nevada Las Vegas 14722, Las Vegas, Nevada, USA; 4 Department of Molecular and Cell Biology, University of Connecticut 7712, Storrs, Connecticut, USA; 5 Gregory Fleming James Cystic Fibrosis Research Center, University of Alabama at Birmingham 9968, Birmingham, Alabama, USA; Indiana University Bloomington, Bloomington, Indiana, USA

**Keywords:** *Pseudomonas aeruginosa*, type III secretion system, T3SS, infection, intracellular replication, corneal cells, *Vibrio fischeri*, reporter plasmid

## Abstract

*Pseudomonas aeruginosa* is known to infect both phagocytic and non-phagocytic host cells, where it exhibits a complex intracellular lifestyle strategy for host survival that is governed by the expression and regulation of its type III secretion system (T3SS). To better understand such complexities in the context of niche bacterial populations in *P. aeruginosa* and other gram-negatives, here we construct and characterize a novel and versatile dual-reporter plasmid, Constitutive GFP, Variable mScarlet-I (pCG-VmS), whose utility proves useful for longitudinal assays of transcriptional reporter activity *in vitro*, *ex vivo*, and in whole animals. As an added utility, we show that pCG-VmS can also be used for translational fusions to monitor bacterial cellular protein localization. We then exploit pCG-VmS to visualize *P. aeruginosa* T3SS expression in a human corneal cell line that identifies a distinct intracellular “quiescent” population, where the T3SS is never expressed. An *ex vivo* murine eye infection model is then used to verify the existence of the two ‘T3SS-on” versus ‘T3SS-off’ expression profiles in infected tissues. In sum, pCG-VmS allows for novel spatiotemporal insights into *P. aeruginosa* protein localization and infection routes and can be tailored to other plasmid backbones amenable to different gram-negative bacteria.

**IMPORTANCE:**

In this study, we develop a novel, well-optimized fluorescent dual-reporter system, pCG-VmS, which can be used for both transcriptional monitoring and protein localization. We demonstrate its use with a range of *in vitro* and *in vivo* experiments with *Pseudomonas aeruginosa* and whole-animal imaging of squid colonization with *Vibrio fischeri*. After showing pCG-VmS efficacy and versatility, we use the plasmid to simultaneously visualize the naturally occurring type-3 secretion system (T3SS) active (T3SS-on) and “quiescent” (T3SS-off) populations during intracellular infection of a human corneal epithelial cell line and the murine eye. Taken together, pCG-VmS is well-balanced in its optical traits and can be used for a variety of both *in vitro* and *in vivo* studies to help separate and further interrogate difficult niche spatiotemporal bacterial populations.

## INTRODUCTION

Dual reporters offer a means by which expression can be both monitored and normalized in real time. Several such systems have been developed to monitor bacterial pathogenesis. However useful, when designing a reporter where results can be trusted, many factors must be considered to ensure rigorous and accurate data, including brightness, oligomerization, maturation time, photostability, ample spectral space between excitations and emissions, and above all, fitness cost, the latter of which has historically complicated data acquisition and interpretation (1, 2). Due to these considerations, most of the available imaging tools for a particular pathogen come with one or more of the aforementioned issues whose results might yield dubious data. However, with the right combinations of fluorophores, novel observations can be accurately visualized and measured.

One pathogen where multi-colored reporter systems are especially useful is the gram-negative *Pseudomonas aeruginosa*, given its medical importance and broad range of hosts and tissues that it can infect. Like many pathogens, several fluorophores have been used to track gene and protein expression in *P. aeruginosa* with single-fluorophore reporter systems, for example, to monitor expression of bacterial second messengers or under oxygen-limiting conditions (3, 4). In other cases where two signals are needed, fluorescently tagged small molecules or fluorescently conjugated antibodies can be used to detect bacterial surface structures (5, 6). More recently, dual (7, 8) and even triple-reporter systems (9) in *P. aeruginosa* and other bacterial pathogens seem to have coalesced into red- and green-emitting versions, in which a GFP variant coupled and a red fluorescent monomeric mScarlet-I variant were chosen, likely for their lack of spectral overlap and other aforementioned attributes. The advantage of such systems has enabled researchers to identify *bona fide* expression and quantifications of subpopulations that otherwise could previously only be anecdotally observed, or not observed at all. In one example, a group was able to visualize the stochastic expression of extracellular biopolymer populations (R-bodies) in only 0.26% of the total population (8).

For many pathogens, including *P. aeruginosa*, the T3SS is crucial for acute infection success (10). Unlike most T3SSs, which export many different effectors (e.g., Salmonella sp.) (11), the *P. aeruginosa* T3SS only exports three major effectors (ExoSTY), and sometimes a fourth (ExoU), which are used to enter host cells, escape the host phagolysosome, and survive the assaults therein (10, 12). Despite its reliance on these effectors, an accurate spatiotemporal assessment of the T3SS usage during infection remains largely unmapped. Previous studies have used a combination of T3SS deletion mutants and reporters that rely on GFP overexpression (using a derivative of the popular pBAD vector), which showed *P. aeruginosa* trapped within the host vacuole when T3SS expression was deleted (13, 14). To enable the *natural* spatiotemporal induction of T3SS expression, we exploited the new dual-reporter plasmid pCG-vmS.

pCG-VmS utilizes constitutive sfGFP and inducible mScarlet-I signals, which are balanced for similar photostability and maturation time, are monomeric, and provide adequate signal for detection. pCG-VmS is first tested for its utility as a transcriptional and translational reporter *in vitro* using a battery of assays. We then demonstrate similar systems using these two fluorophores (sfGFP and mScarlet-I) can be engineered successfully in a different gram-negative bacterium, where we show differential expression of a target gene during colonization of the optically accessible Hawaiian bobtail squid *Euprymna scolopes* by *Vibrio fischeri*. pCG-vmS was then used to interrogate T3SS expression first in cultured corneal cells, where we could clearly observe both the minor T3SS-off population and the T3SS-on population. These two populations were then shown in a whole murine eye infection model. Taken together, we introduce a versatile dual-reporter system that can be used to visualize distinct bacterial populations and has thus far produced novel insights into the utilization of the T3SS during corneal infection.

## RESULTS

### Design and characterization of pCG-VmS

To develop a plasmid system that would have wide utility in monitoring expression, at either the transcriptional or translational level, we utilized the plasmid pCC21 as a backbone structure (15). pCC21 maintains a low copy number in *E. coli* (approximately 5–10 copies per cell) through the pBBR1 origin and has been shown to be low to medium copy and well maintained in *Pseudomonas putida* (average of 30 copies per bacterial cell) (16). Moreover, the pBBR1 origin exhibits broader host utility (17) than more *Pseudomonas* sp.-specific origins such as ColE1/pRO1600 (18). For reporter gene output, after an initial fluorescent protein (FP) screening process, the engineered monomeric red fluorescent protein (RFP) mScarlet-I was selected due to its inherent high brightness and low background fluorescence (resulting from its red-shifted emission wavelength), which allows for increased sensitivity when compared to other more blue-shifted FPs (19). The mScarlet-I variant of mScarlet also possesses a more rapid maturation time when compared to other RFP variants (20), making it a preferred candidate for a reporter gene that can maintain sensitivity while enabling rapid detection. Superfolder GFP (sfGFP) was then selected as a constitutive fluorescent signal due to its closer maturation time to mScarlet-I in microorganisms (sfGFP ~10 min versus mScarlet-I ~30 min) (21), as well as enhanced brightness (20). Despite its longer folding time compared to sfGFP, mScarlet-I currently exhibits the fastest maturation time for an RFP. We chose a green-red combination with sfGFP and mScarlet-I because they have little spectral overlap and can therefore be excited and detected at or near their peak wavelengths, ensuring low background or “bleed though” signals and the highest intensity for detection (Fig. S1). Finally, sfGFP and mScarlet-I were selected for their comparable photostability (sfGFP *t*_1/2_ = 208.26 s and mScarlet-I *t*_1/2_ = 225.0 s). Thus, their bleaching times are relatively similar, which is especially important for longitudinal microscopy imaging techniques.

**Fig 1:**
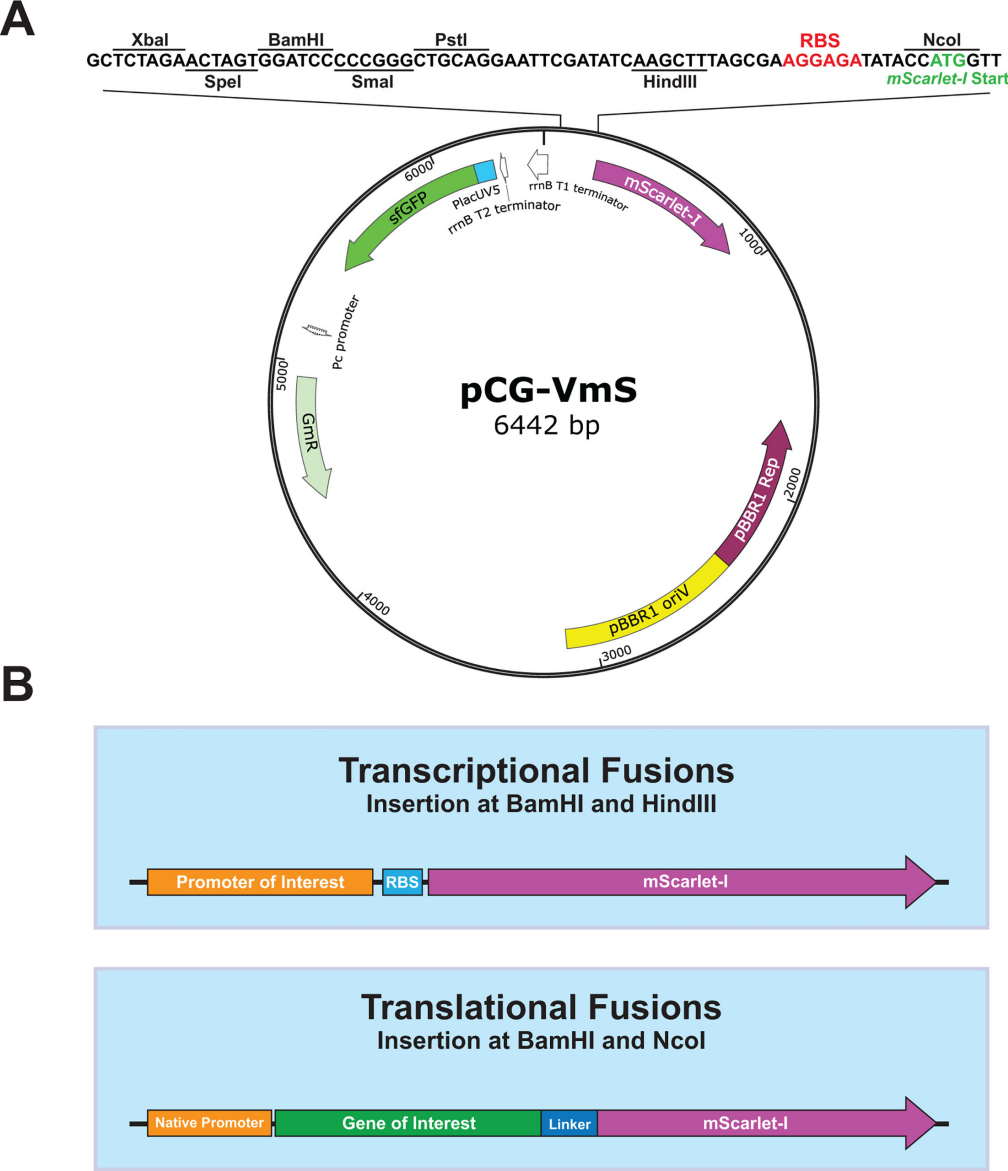
pCG-VmS design. (**A**) MCS and plasmid map of pCG-VmS. The ribosome binding site (RBS) and mScarlet-I start codon are highlighted in red and green, respectively. The plasmid map was generated using SnapGene. (**B**) Schematic of transcriptional and translational fusions that can be made depending on which restriction enzymes are utilized for insertion.

The plasmid was also designed for easy insertion of promoters upstream of mScarlet-I (with its associated RBS) to generate transcriptional fusions, utilizing several single-cut restriction sites (*Xba*I, *Spe*I, *BamH*I, *Sma*I, *Pst*I, and *Hind*III) (Fig. 1A and B). For translational fusions, the RBS can be removed by digestion with *Nco*I (also a single-cut site in the vector) and another selected upstream restriction enzyme site. The gene of interest and its upstream regulatory region can then be inserted in frame using *mScarlet-I* with a 3′ linker region, since the *Nco*I site contains the mScarlet-I start codon, to create a C-terminal mScarlet-I fusion protein (Fig. 1B). In addition, Rho-independent *rrnB* T1 and T2 terminators (22) were inserted upstream of the MCS to prevent transcription from initiating upstream of the *mScarlet-I* promoter.

To constitutively express sfGFP at levels amenable to most detection needs, a synthetic *lac*-based promoter, P_A1/04/03_ (23, 24), was originally chosen for its ability to drive high levels of protein expression in gram-negative bacteria and for its previous use as a constitutively expressed promoter for fluorophores in *P. aeruginosa* (8, 24, 25), including in previously used dual-reporter systems (8). However, preliminary testing with this promoter driving sfGFP expression resulted in a disruptive bacterial cell filamentation phenotype—a phenomenon that occurred when mScarlet-I was simultaneously expressed (using the promoter for *exoS*) with constitutive sfGFP (Fig. S2, Movie S1). Alternatively, a subpopulation of bacteria was observed that suppressed expression of sfGFP (seen within the same field containing filamentous bacteria in Fig. S2) (see Box 2 and Movie S1). Indeed, it has been previously observed that, in *P. aeruginosa*, high expression of two fluorophores can lead to unwanted growth or virulence defects, as well as mutagenesis and/or loss of the vector to suppress expression of one or both fluorophores (26). To circumvent this problem, the P_A1/04/03_ promoter was exchanged for the weaker *lacUV5* promoter, which also exerts its expression independent of CRP control (23). After this alteration, the sfGFP intensity was reduced, alleviating the cell filamentation phenotype that occurred with simultaneous mScarlet-I expressed from a strong promoter (Movie S1). The finalized plasmid was named pCG-VmS (Constitutive GFP, Variable mScarlet-I) (Fig. 1A).

### pCG-VmS assessment during *in vitro* growth

**Fig 2:**
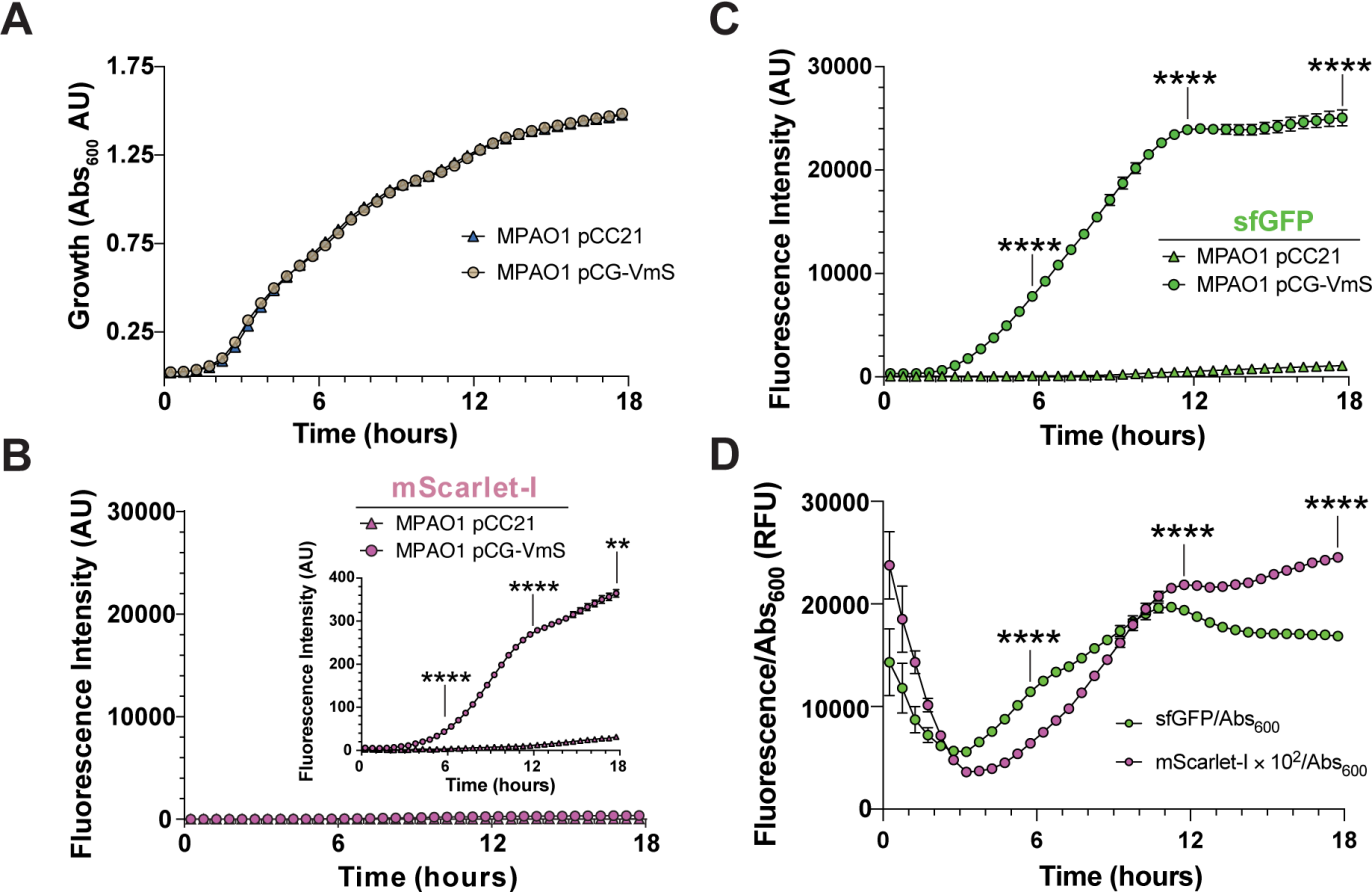
Characterization of pCG-VmS during planktonic growth. *P. aeruginosa* MPAO1 harboring pCG-VmS or the non-fluorescent control plasmid pCC21 was grown in LB medium with antibiotics for 18 h. (**A**) Absorbance at 600 nm, (**B**) raw mScarlet-I signal, and (**C**) raw sfGFP fluorescence signal over time is shown. Inset in panel B: graph with alternative y-axis scale to highlight the very slight mScarlet-I background expression in pCG-VmS despite the absence of a promoter. (**D**) mScarlet-I and sfGFP signals were calculated as a ratio and then normalized using the absorbance at 600 nm. Normalization of mScarlet-I and sfGFP signals to Abs_600_ results in a similar “S”-shaped curve, which could be due to fluorescence dilution and/or fluorophore maturation. The mScarlet-I signal was multiplied (×10^2^) to make the curve shapes comparable, due to the low mScarlet-I signal in the absence of a promoter. Statistics: A two-way ANOVA with Šídák’s multiple comparisons was applied. **, *P* < 0.01; ***, *P* < 0.001; ****, *P* < 0.0001. AU, arbitrary units; RFU, relative fluorescence units.

When compared to its non-fluorescent parent plasmid pCC21, pCG-VmS caused no observable deleterious effects on the growth of WT *P. aeruginosa* MPAO1 (Fig. 2A). As stated before, the low background fluorescence at red-shifted wavelengths allows for increased sensitivity of RFPs, such as mScarlet-I (27). However, cells harboring pCG-VmS did exhibit some detectable mScarlet-I fluorescence throughout 8 h of growth compared to the background levels of the non-fluorescent control (Fig. 2B), indicating that a low, basal level of mScarlet-I signal is produced despite the introduction of the terminators. Robust sfGFP signal, well above the background intrinsic fluorescence (produced by the bacteria and media), was also seen (Fig. 2C; ~100-fold increase in sfGFP channel fluorescence after 8 h compared to the non-fluorescent control). In addition, the raw fluorescence values of the sfGFP signal closely mimicked the growth of the cells when measured by the absorbance at 600 nm (Fig. 2A and C). When the sfGFP signal was normalized to the OD_600_ value, a noticeable decrease in the fluorescence intensity was seen as the cells entered the logarithmic growth phase (Fig. 2D). One likely explanation for this phenomenon is dilution of the sfGFP molecules as the cells divide rapidly (28) as well as a slight delay in fluorescence of newly synthesized sfGFP, which must first mature. Similarly, a decrease in fluorescence density was also seen when monitoring the mScarlet-I signal (Fig. 2D). Since both FPs should theoretically be equally affected by this decrease, normalization of the mScarlet-I signal to the sfGFP signal would therefore be a preferred form of normalization rather than the optical density—a superiority that is already well supported (29).

### Utility as a transcriptional reporter in planktonic and biofilm-forming *P. aeruginosa*

**Fig 3:**
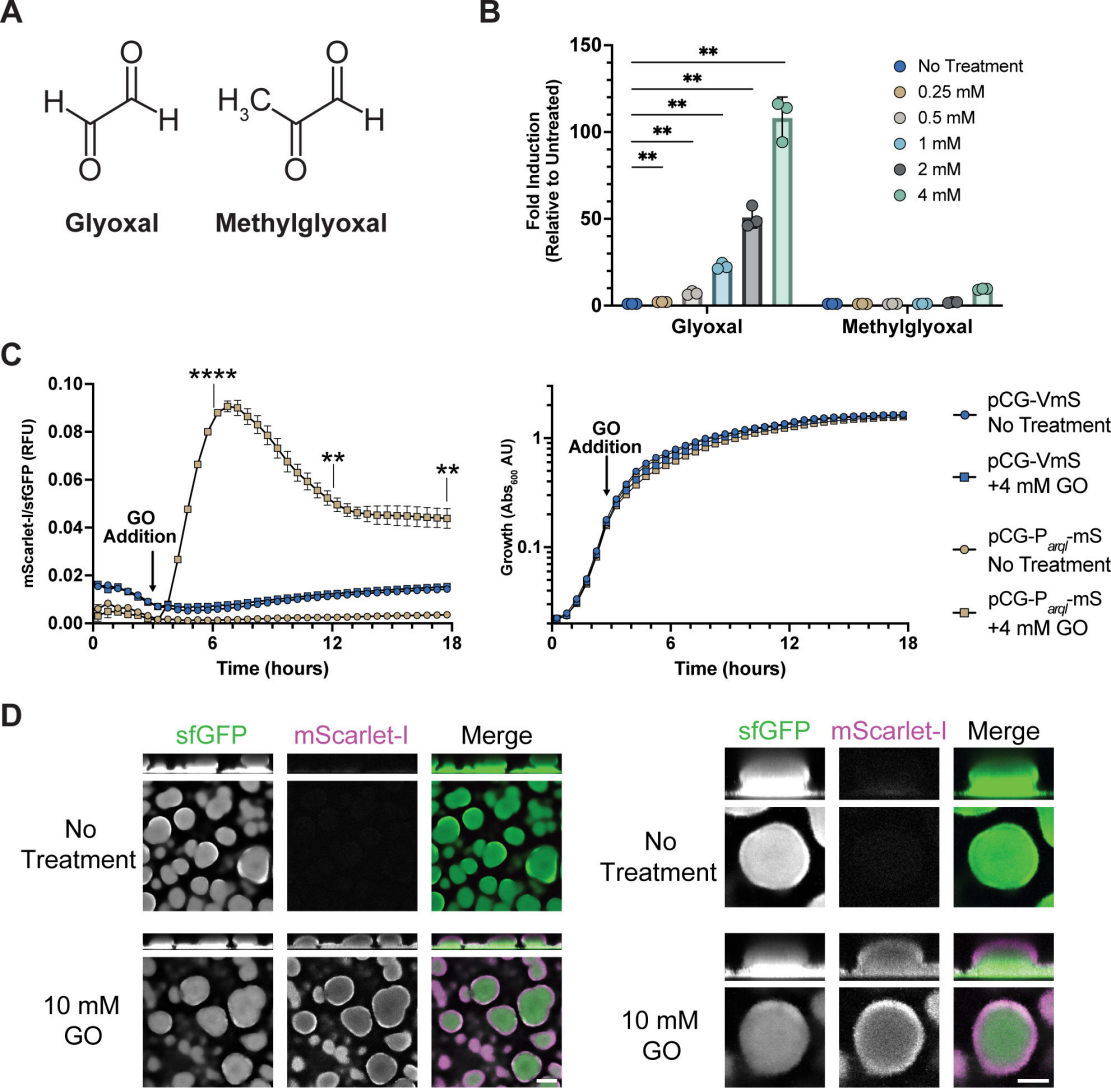
pCG-VmS transcriptional reporter utility. (**A**) Chemical structures of GO and MGO. (**B**) Dose response of pCG-P*_arqI_*-mS to GO and MGO at 3 h post-addition. Values are expressed as mScarlet-I/sfGFP relative to the no treatment control. (**C**) (Left) Normalized mScarlet-I fluorescence signal to constitutive sfGFP signal and (right) growth according to absorbance at 600 nm of pCG-P*_arqI_*-mS after 4 mM GO was added at 3 h post*-P. aeruginosa* inoculation. Growth curves on the right are the same, indicating that the fluorescence data on the graph on the left is not influenced by growth defects from expressing the fluorophores. (**D**) Induction of the *arqI* promoter by 10 mM GO treatment in biofilms grown in a flow cell. Representative image of a field of biofilm aggregates (left) with an enlarged image of one aggregate (right). For each set of images, the top view is the side (XZ)-view, and the bottom is the cross-sectional (XY)-view. Scale bar = 100 µm (left) or 50 μm (right). AU, arbitrary units; RFU, relative fluorescence units. Statistics: *, *P* < 0.05; **, *P* < 0.01; ***, *P* < 0.001 by a one-sample *t* test.

We recently demonstrated that *arqI-gloA2* operon transcription is tightly controlled and specifically induced upon exposure of *P. aeruginosa* to the aldehyde glyoxal (GO), but not by other aldehydes such as methylglyoxal (MGO) (15) (Fig. 3A). Due to this tight control and inherent specificity, we exploited the *arqI* promoter to test the ability of pCG-VmS to perform as a transcriptional reporter. One hundred forty-five base pairs of DNA sequence upstream of the *arqI* start codon were cloned into pCG-VmS to create a transcriptional reporter for the *arqI-gloA2* operon, yielding the plasmid pCG-P*_arqI_*-mS. In line with our previous work, we found that transcriptional induction from this promoter was highly controlled and specific to GO (Fig. 3B). pCG-P*_arqI_*-mS showed a high level of induction, with an approximately 100-fold change in normalized reporter activity at 4 mM GO when compared to untreated control. Notably, our previous studies utilizing P*_arqI_* transcriptional fusions to a fluorescent reporter found only a 10-fold increase in promoter activity upon treatment with 4 mM GO, suggesting that when normalized to sfGFP signal, the mScarlet-I signal from the dual pCG-P*_arqI_*-mS system may exhibit increased sensitivity due to lower background interference when compared to previous, single-reporter constructs (15). After GO addition, reporter activity increased rapidly, with detectable levels of mScarlet-I fluorescence above baseline appearing 30 min post-GO addition and increasing over a period of 3.5 h (Fig. 3C, left). Of note, insertion of the *arqI* promoter reduced the mScarlet-I signal below the level of the empty plasmid control, suggesting repression of the promoter by an unknown transcription factor and/or decreased polymerase read-through (Fig. 3C, left). Important for pCG-VmS utility, we could not detect any growth defects even after the highest levels of mScarlet-I signal were achieved, indicating negligible cytotoxicity (Fig. 3C, right).

### Use of pCG-VmS to observe promoter expression in biofilms using a flow cell

We next sought to investigate the use of pCG-P*_arqI_*-mS in more complex three-dimensional environments by exploiting flow cell technology. WT MPAO1 biofilms were grown in a flow cell in 1% TSB media for 96 h before the addition of 10 mM GO. After 3 h of GO incubation, the mScarlet-I signal increased, with a noticeable heavier induction around the periphery of the biofilm (Fig. 3D). This suggests that GO may have a limited ability to penetrate the biofilm mass and confirms the utility of this reporter plasmid for imaging spatiotemporal gene expression within self-contained flow cell systems and/or biofilms.

### Use of pCG-VmS to visualize promoter expression at the single-cell level with flow cytometry

**Fig 4:**
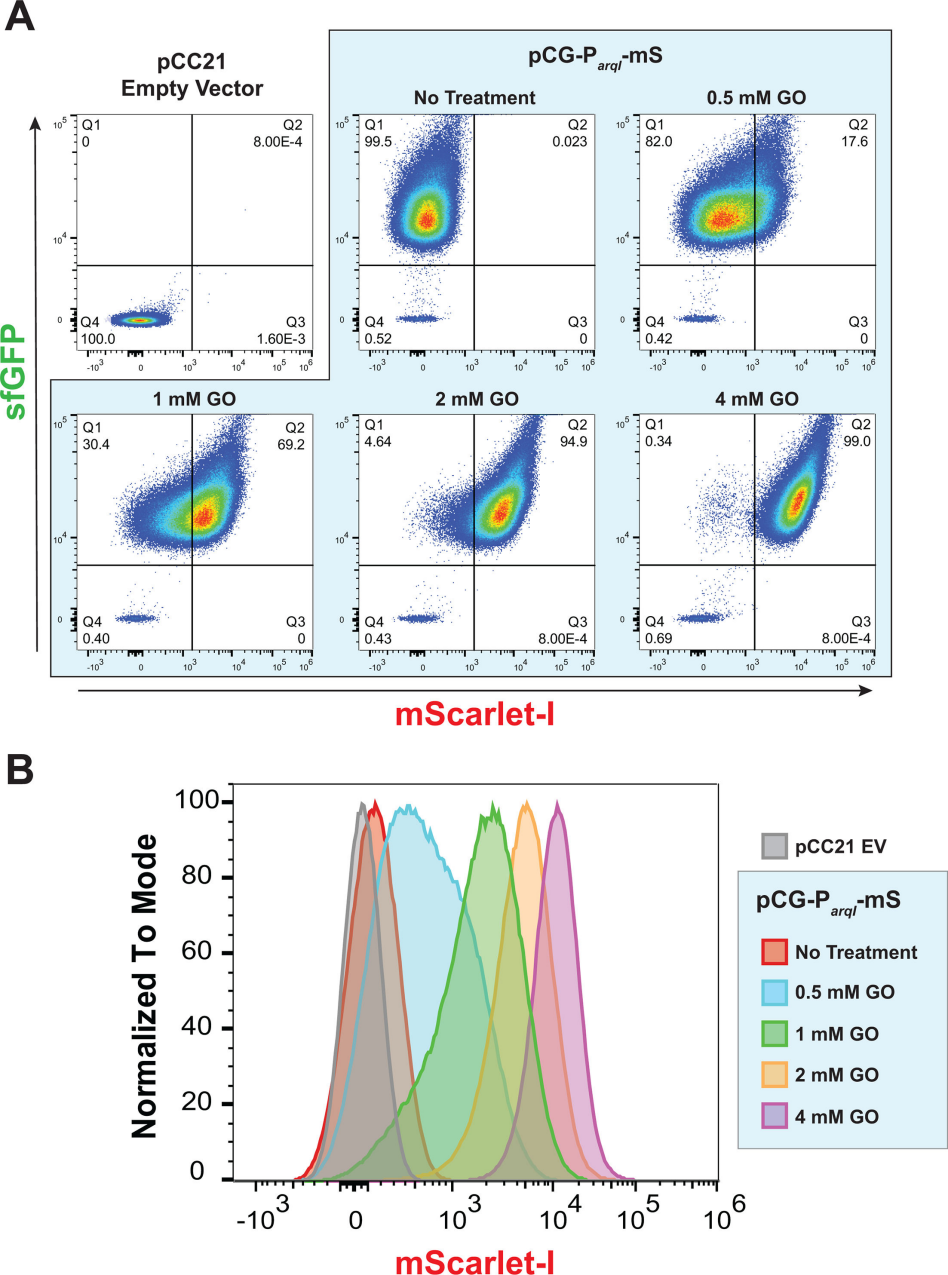
Population dynamics of *arqI* promoter activation in response to GO. (**A**) Pseudo-colored dot plots of mScarlet-I expression upon increasing concentrations of GO added to MPAO1 pCG-P*_arqI_*-mS cells depicting sfGFP-positive population and mScarlet-I induction by GO. (**B**) Flow cytometry histogram of data presented in panel A. Populations are normalized to mode. One representative of three biological replicates is shown. MPAO1 pCC21 was used as a non-fluorescent control. EV, empty vector.

To visualize the dynamics of *arqI-gloA2* promoter induction at the single-cell level, flow cytometry was used. Relative to the non-fluorescent parent plasmid pCC21, the pCG-P*_arqI_*-mS harboring strain exhibited robust sfGFP fluorescence in roughly 99.5% of cells analyzed by flow cytometry (Fig. 4A). Conversely, in the absence of GO, minimal levels of mScarlet-I signal were seen, indicating that there is some expected basal level of *arqI* promoter activity under standard culture conditions (Fig. 4B; red peak). When increasing concentrations of GO were added, the *arqI-gloA2* promoter showed a graded response (Fig. 4A and B). The addition of 0.5 mM GO resulted in a wide distribution of mScarlet-I signal, suggesting differential induction occurring across the population of bacteria (Fig. 4B, blue peak). As the concentration of GO increased, the population exhibited increasingly homogeneous expression of mScarlet-I, as indicated by a sharper peak, culminating in 95% induction at 2 mM and 99% induction at 4 mM GO (Fig. 4B).

### pCG-VmS utility as a translational reporter for protein cellular localization

**Fig 5:**
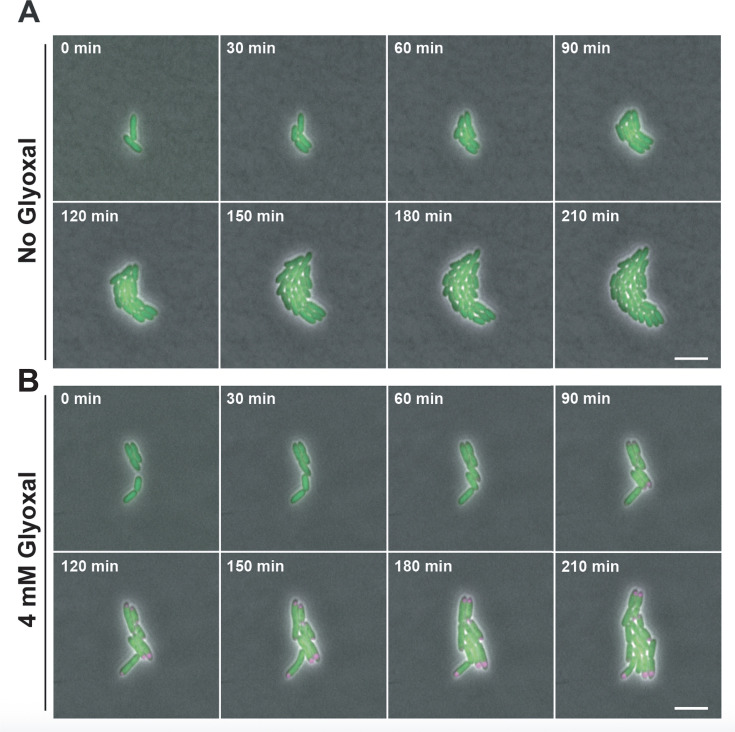
Polar localization of the ArqI-mScarlet-I fusion protein following induction by GO. (**A**) Time-lapse microscopy of MPAO1 pCG-P*_arqI_*-ArqI-mS cells cultured to mid-logarithmic phase before transfer to a 1.5% agarose pad without or (**B**) with 4 mM GO added (bottom panel) for imaging. Scale bar = 5 µm. Green, constitutive sfGFP; red puncta at the flagellar pole, mScarlet-I translational fusion to ArqI.

Our previous work determined that sfGFP fusions to ArqI WT protein hexamer migrated to the flagellar pole upon GO induction, whereas mutants that resulted in ArqI dimers did not (15). The biological consequences of this migration have yet to be determined. To test the utility of pCG-VmS for use with translational fusions and subcellular protein localization, the *arqI* promoter and coding sequences were inserted in-frame with *mScarlet-I* with a linker to form an [ArqI]-[mScarlet-I] fluorescent fusion protein. WT MPAO1 cells harboring this fusion plasmid were spotted onto 1.5% agarose LB pads and imaged over the course of 3.5 h. In the absence of GO, no localization or robust red fluorescent signal was seen, only the constitutive sfGFP signal that outlined the shape of dividing bacteria (Fig. 5A). However, as we previously observed with our sfGFP fusion to ArqI (15), upon GO induction, the ArqI-mScarlet-I WT hexamer fusion protein clearly localized to the flagellar pole in a subpopulation of bacteria (Fig. 5B). These results show that pCG-VmS can also be used to detect bacterial cell protein localization using translational fusions.

### *In vivo* imaging of *V. fischeri*-colonized juvenile *E. scolopes* squid

Due to the low background fluorescence and high brightness of the mScarlet-I reporter, we hypothesized that the pCG-VmS reporter cassette would allow for robust *in vivo* imaging in an optically accessible animal model. One animal with a relatively optically clear body is the Hawaiian bobtail squid, *Euprymna scolopes*, whose symbiotic light organ is colonized by the bacterium *V. fischeri* (30, 31). As a result of the light organ properties, it is possible to achieve spatiotemporal visualization of *Vibrio fischeri* reporter constructs as the bacteria colonize the light organ. To capture these dynamics, dual fluorescence vectors have been utilized for *in vivo* imaging of colonized juvenile *E. scolopes* squid before (32, 33). To determine if our dual-reporter system would work in *V. fischeri*, we transferred the fluorescence cassette from pCG-VmS into the *Vibrio*-specific plasmid, pVSV105 (32), creating pVSV105-CG-VmS. Due to differences in sfGFP expression in *V. fischeri*, we utilized the constitutive P_A1/04/03_ promoter (34) in the pVSV105-CG-VmS plasmid to increase the sfGFP signal to the required, more robust levels.

**Fig 6:**
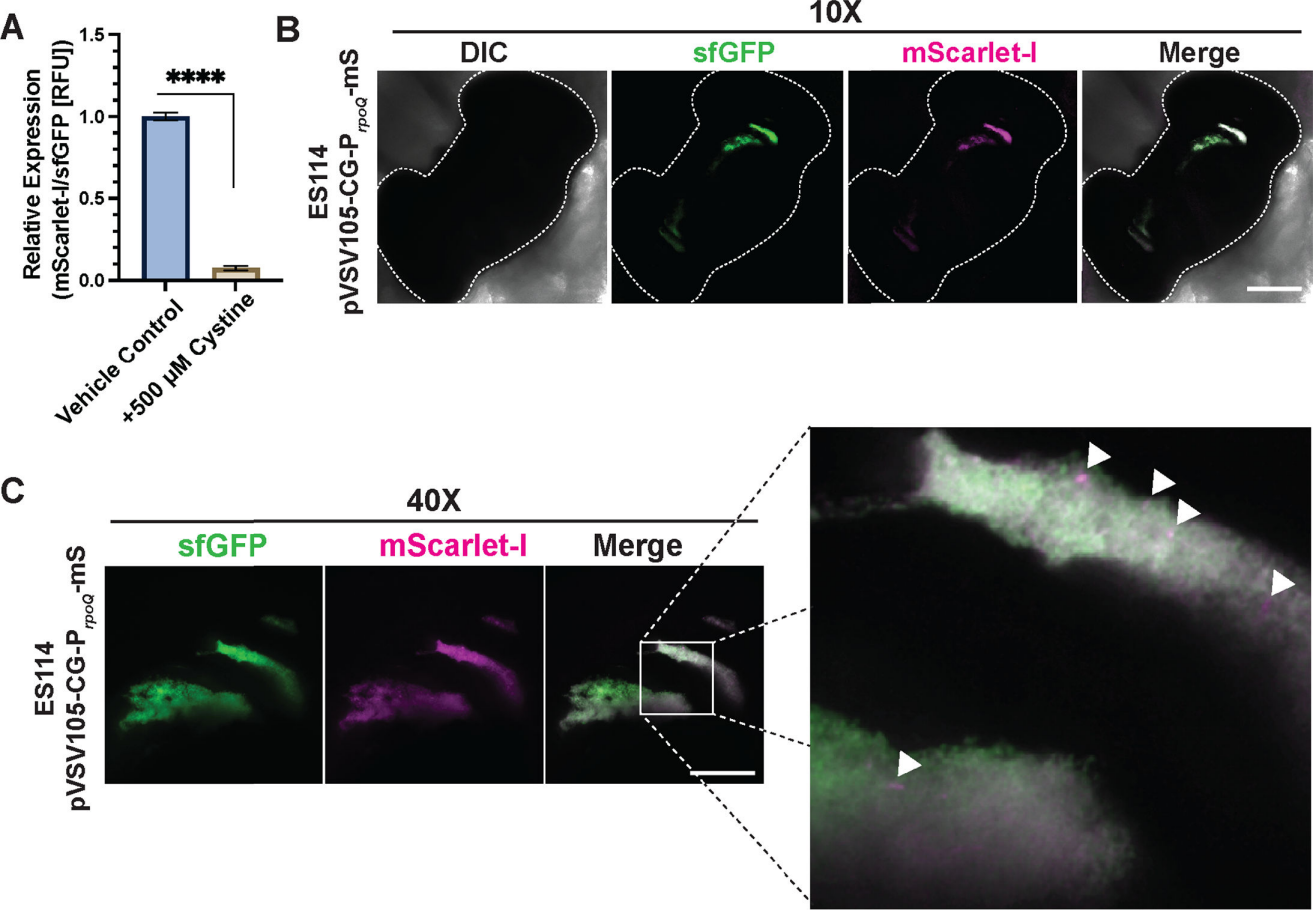
*In vivo* imaging and *in vitro* induction of pVSV105-CG-VmS-derived plasmids in *V. fischeri*. (**A**) *In vitro* repression of pVSV105-CG-P*_cysK_*-mS by the addition of cystine to the growth media (*n* = 2 biological replicates). ****, *P* < 0.0001 by a one-sample *t*-test. 10× (**B**) and 40× (**C**) *in vivo* imaging of ES114 pVSV105-CG-P*_rpoQ_*-mS colonized juvenile *E. scolopes* bobtail squid showing colocalization of sfGFP and mScarlet-I signal in light organ crypt spaces. The light organ and ink sac are outlined with a dotted line in panel B. Scale bars = 150 µm (B) and 50 μm (C). Arrows point to areas of concentrated mScarlet-I expression.

After creating pVSV105-CG-VmS, we sought to first verify its utility in *V. fischeri* by examining its expression in culture (*in vitro*). We constructed a transcriptional reporter using the promoter of *cysK* (pVSV105-CG-P*_cysK_*-mS), which is highly repressed in *V. fischeri* in the presence of cystine (35). When grown in minimal media supplemented with cystine, an ~10-fold decrease in signal was detected compared to media without any cystine supplementation (Fig. 6A). This decrease in reporter activity is in agreement with previous results of *cysK* promoter activity (35) and highlights the utility of pVSV105-CG-VmS as a viable *in vitro* transcriptional reporter. We then tested pVSV105-CG-VmS as a reporter system for whole-animal imaging. For these experiments, we used the promoter for the alternative sigma factor *rpoQ* (plasmid pVSV105-CG-P*_rpoQ_*-mS) for two reasons: (i) *rpoQ* encodes an unusual sigma factor whose overexpression impacts motility, luminescence, and chitinase activity (phenotypes that are relevant to the interaction of *Vibrio fischeri* with its symbiotic host) (36), and (ii) transcription from the *rpoQ* promoter is controlled by the important LitR transcription factor (37, 38).

Hatchling *E. scolopes* were colonized with *V. fischeri* pVSV105-CG-P*_rpoQ_*-mS for 24 h prior to fixation and fluorescence microscopy. Robust sfGFP signal was observed within the light organs of colonized hatchlings, indicating successful colonization by the reporter strain. In addition, mScarlet-I signal was observed and colocalized with the sfGFP signal, verifying transcriptional activation of the *rpoQ* promoter by *V. fischeri* within the squid light organ (Fig. 6B). P*_rpoQ_* activity appeared to be expressed across the entire colonized area, with additional distinct puncta of high reporter signal present, as can be seen in the merged channel image (Fig. 6C). These pockets of elevated RpoQ activity could represent initial areas of colonization driven by RpoQ gene regulation. Taken together, these results show that the sfGFP/mScarlet-I combination/cassette in the original plasmid for *P. aeruginosa* can be engineered for use in other gram-negatives and is a good selection of compatible fluorophores to enable longitudinal imaging in whole animals.

### Visualization of T3SS-positive (on) and -negative (off) populations during intracellular infection

*P. aeruginosa* has the ability to invade epithelial cells (39) and establish a cytoplasmic niche (12, 26, 40). After initial internalization, the pathogen activates the T3SS to help escape the vacuole (26) and freely replicates inside the cytoplasm until subsequent host cell death (14). The *P. aeruginosa* expression of the T3SS (i.e., “T3SS-on” population) has been established using pJNE05—a single-fluorophore plasmid in which the promoter of the T3SS toxin *exoS* drives GFP expression. On the other hand, another population of invading *P. aeruginosa* has been observed that does not appear to utilize their T3SS, aptly dubbed the “T3SS-off” population. This population was first seen through the use of another single reporter that monitored the expression of *cdrA* gene, which highlighted a niche population that survived in the vacuole but, conversely, ultimately failed to enter the cytoplasm (13). Importantly, CdrA is a cyclic-di-GMP-dependent biofilm-inducing protein and when it is activated, T3SS expression is usually “turned off.” Thus, here, the inference was that if CdrA was expressed inside a vacuole, although not directly measured, the T3SS should be off. With these studies, all *P. aeruginosa* bacteria could also be labeled using a *P. aeruginosa*-specific antibody; however, the cells had to be fixed which prevented spatiotemporal data acquisition. A more recent remedy for this issue has exploited an arabinose-induced GFP to visualize all intracellular bacteria and has been used to measure spatial quantities of GFP + puncta to quantify only observably smaller, occupied “T3SS-off” vacuoles (41). Nevertheless, while arabinose-inducible GFP vectors are a powerful means to control GFP expression, they have been found to actually suppress T3SS activity (42) and have other general limitations in *P. aeruginosa* (43). Therefore, to address these issues, we exploited pCG-VmS to visualize all bacteria and native expression of the *exoS* promoter simultaneously during *P. aeruginosa* intracellular replication. For these studies, we transcriptionally fused the same *exoS* promoter (P*_exoS_*) DNA to mScarlet-I in pCG-VmS, yielding the new T3SS reporter pCG-P*_exoS_*-mS.

**Fig 7:**
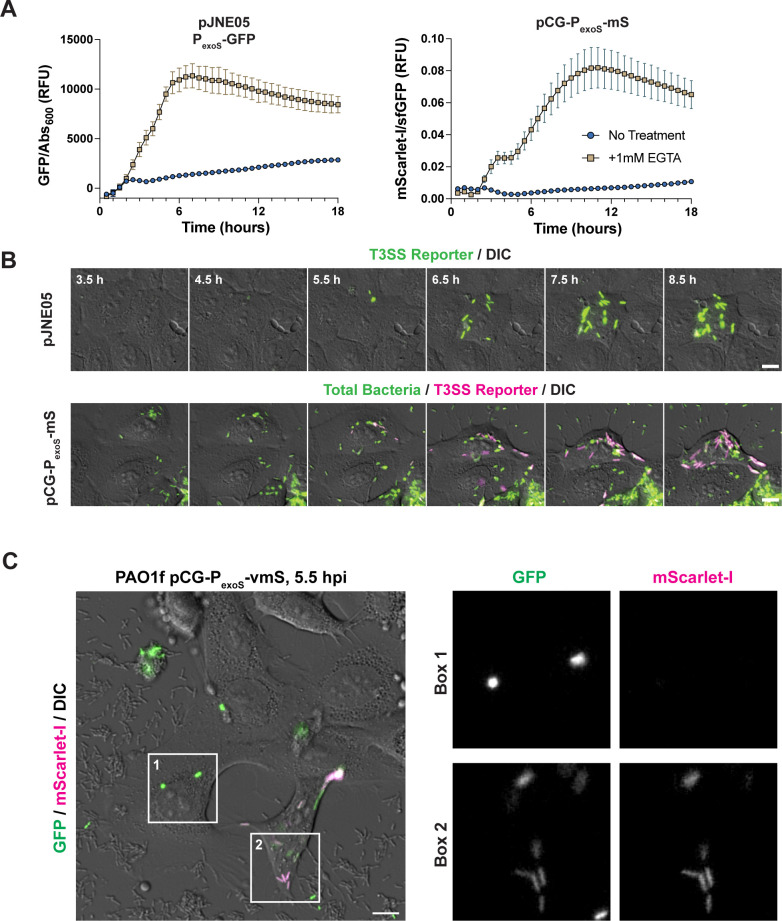
Induction of pCG-P_exoS_-mS by EGTA and infection of corneal epithelial cells. (**A**) Normalized fluorescent signals of EGTA-mediated induction of the *exoS* promoter in PAO1f harboring the classic single-fluorophore plasmid pJNE05 to show *exoS* promoter induction in comparison to OD (left) and the dual-reporter pCG-P*_exoS_*-mS, where normalization is accomplished by the constitutive sfGFP signal (right). 1 mM EGTA addition activates the T3SS *exoS* promoter. (**B**) *In vitro* infection of hTCEpi corneal epithelial cells comparing the kinetics of T3SS activation between pJNE05 (above) and pCG-P*_exoS_*-mS (below) in PAO1f. Scale bar = 10 µm. (**C**) *In vitro* infection of hTCEpi corneal epithelial cells invaded by PAO1f with pCG-P*_exoS_*-mS shown at 5.5 h post-infection. At 3 h post-infection, extracellular bacteria were killed with amikacin and polymyxin B, which diminishes constitutive sfGFP fluorescence of extracellular bacteria by 5 h post-infection. Populations of intracellular T3SS-off (box 1) and T3SS-on (box 2) bacteria are shown. RFU, relative fluorescence units.

We first directly compared the aforementioned single-fluorophore reporter pJNE05 plasmid with our new dual-reporter pCG-P*_exoS_*-mS vector. Results showed similar reporter induction kinetics when T3SS was induced using the calcium-chelating agent EGTA, as measured by the mScarlet-I/sfGFP ratio over time (Fig. 7A). As expected, we observed a lower background signal with the pCG-P*_exoS_*-mS data, likely due to the more accurate method of utilizing a second FP signal (sfGFP) to normalize. In early log phase (lag phase to approximately OD_600_ 0.3), a slight delay in reporter activity was seen with pCG-P*_exoS_*-mS, which could be due to the marginally longer maturation time of mScarlet-I (about 2-fold) compared to GFP (20). To assess the utility of pCG-P*_exoS_*-mS during infection, we exposed telomerase-immortalized human corneal epithelial cells (hTCEpi cells) to WT PAO1f harboring either pJNE05 or pCG-P*_exoS_*-mS. T3SS activation could be visualized within 4.5 h post-infection, with expansion of the T3SS-on population being comparable between the two strains (Fig. 7B; Movie S2). However, the use of our pCG-P*_exoS_*-mS plasmid also allowed visualization of the expanding T3SS-off populations when extracellular fluorescent bacteria were cleared with the addition of polymyxin B (Fig. 7C; Movie S3). As noted in past publications (13), we observed that some T3SS-off bacteria resided inside the same cells as expanding T3SS-on bacteria. Detection of this previously untraceable T3SS-off population using pCG-P*_exoS_*-mS verifies evidence for the existence of this niche, quiescent population from prior studies (12) and, importantly, paves the way for its accurate quantitation and investigation of how it might dictate the vacuolar exit process.

### Infection of murine corneas with the pCG-P*_exoS_*-mS T3SS reporter

**Fig 8:**
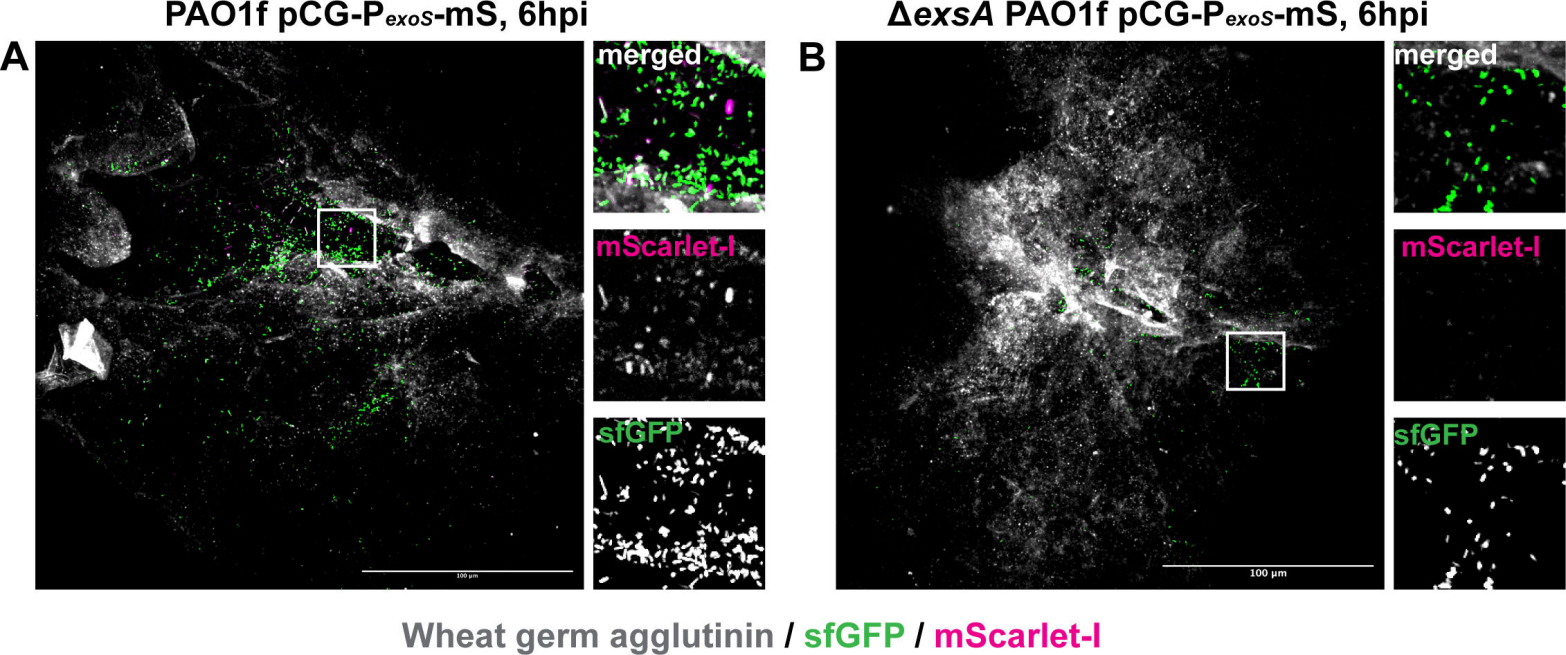
Use of pCG-P*_exoS_*-mS during *ex vivo* mouse cornea infection. Confocal fluorescence microscopy of scratch and tissue-blotted *ex vivo* 8- to 12-week-old C57/BL6 mouse cornea infection with (**A**) wild-type PAO1f pCG-P*_exoS_*-mS and (**B**) ∆*exsA* (T3SS-null) PAO1f pCG-P*_exoS_*-mS taken at 6 h post-infection. Insets show populations of bacteria interacting with corneal epithelium from white boxed areas. Scale bar = 100 µm. Labeled agglutinin appears white. *P. aeruginosa* bacteria appear green, and if the *exoS* promoter is active, a magenta signal is visible.

It has long been understood that corneal abrasion can lead to keratitis (44). However, how the T3SS is involved in these infections through spatiotemporal expression has not been examined. To begin to answer this question, we used our pCG-P*_exoS_*-mS reporter strain to infect excised mouse eyes and performed spatiotemporal imaging after a 4-hour infection with eyes that had been wounded (scratched). As a control, we used a *∆exsA* strain of *P. aeruginosa*, which is unable to make the T3SS, resulting in a loss of ability to escape the host vacuole (12, 13). Results showed that T3SS expression continued to maintain a heterogeneous T3SS-on and T3SS-off bimodal population (Fig. 8; Movie S4 and Movie S5). Similar to our cell-based corneal infections in Fig. 7, whole *ex vivo* imaging of the wounded eye showed a mixed population of T3SS-on and T3SS-off populations. Taken together, these studies show for the first time that *P. aeruginosa* maintains a heterogeneous T3SS-dependent population during both *in vitro* and *in vivo* infection of corneal epithelial cells.

## DISCUSSION

Here, we developed and characterized pCG-VmS, a dual-fluorophore transcriptional and translational reporter plasmid for use in gram-negative bacteria, using *P. aeruginosa* and *V. fischeri* as proof-of-concept microorganisms. Utilizing the GO-responsive *arqI* promoter (15), we established that our plasmid provides robust sensitivity as a transcriptional reporter and allows visualization of promoter activity in complex environments, such as within a biofilm. We also demonstrated that pCG-VmS could be used to map the cellular localization of proteins without having to add additional dyes to highlight the volume of the bacterial cell and provide context to its perimeter. The constitutive sfGFP signal allowed for reporter activity normalization, as well as visualization of viable bacteria during confocal microscopy. Although not shown here, given the high photostability and brightness of the pCG-VmS FPs, the current version of pCG-VmS could be used with Structured Illumination Microscopy (SIM), which demands these enhanced FP attributes. Further, the vector could be easily modified to accommodate the photoswitchable FP versions required for use with stochastic optical reconstruction microscopy (STORM) (e.g., mEos3.2, mMaple3, or PA-mKate) (45).

While we have not constructed a plasmid version to facilitate bioluminescence or Forster resonance energy transfer experiments (BRET and FRET, respectively), one could easily envision modifying pCG-VmS to do so. Alternatively, a third fluorophore could be incorporated into the plasmid which presents a minimum spectral overlap with the other two signals. Indeed, a triple-reporter system has recently been accomplished to monitor complex mixtures of cyclic nucleotides in *P. aeruginosa* cells using mScarlet-I and mGreenLantern as the variable reporter signals and the cyan fluorescent protein SCFP3A as a constitutive readout (9). The fact that both this system and the pCG-VmS system described here utilize mScarlet-I as a readout highlights the superior compatibility of this bright, monomeric fluorophore for bacterial imaging.

In this work, we also demonstrated that the pCG-VmS fluorescence cassette can be versatile by transferring it to different plasmid backbones, which enabled its use in other species of gram-negative bacteria. In our example using *V. fischeri*, transfer of the cassette to a *Vibrio*-specific plasmid allowed for transcriptional reporter activity to be quantified *in vitro* and visualized *in vivo* during colonization of *E. scolopes*. The current plasmid backbone utilized in pCG-VmS, pBBR1, was originally isolated from *Bordetella bronchiseptica* (46), but is usable within a wide array of gram-negative bacterial genera, including *Escherichia, Rhizobium, Pseudomonas, Salmonella, Klebsiella, Agrobacterium, Azospirullum,* and *Serratia* species (46, 47). For these and other species, similar to our *V. fischeri* constructions, it is plausible that different constitutive promoters might be required to “fine-tune” the normalization signal.

Since the pCG-VmS constitutive expression of sfGFP is driven by the *lac*-based promoter *lacUV5*, one could conceive that sfGFP expression could be dampened by an endogenous LacI repressor found in many gram-negative genomes. Nevertheless, we have observed that, despite it possessing an endogenous LacI repressor, *E. coli* DH5α harboring pCG-VmS does exhibit a measurable sfGFP fluorescence signal. This could be due to the stoichiometry of promoter to LacI levels within the cell, which would be in favor of the promoter on multi-copy plasmids. Indeed, this effect has been well established in the literature, with an increase in copy number of promoters leading to a decrease in repression efficiency by LacI (48). Therefore, sufficient sfGFP expression from pCG-VmS may, in fact, be possible even in *lacI*^+^ bacteria. Moreover, the exact activity of the *lacUV5* promoter across different species can vary, as was the case in *V. fischeri* here, which we have anecdotally observed as having lower sfGFP signal compared to that of *P. aeruginosa*. We attribute this phenomenon to the *E. coli* σ^70^-dependent promoter not being efficiently recognized by the cognate RNAP holoenzyme in Vibrio species. An alternative hypothesis is that LacI homologs from different species might have slightly different DNA recognition motifs, which could affect repression when using the heterologous *lacUV5* promoter sequence (49). This problem might be circumvented by utilizing the constitutive P_A1/04/03_ promoter, or a promoter with similar strength that provides higher expression (as was accomplished in *V. fischeri* here), or simply by changing the LacI recognition site in the *lacUV5* promoter. Thus, pCG-VmS and pVSV105-CG-VmS have the potential to be useful in many gram-negative bacterial species but might require fine-tuning expression of sfGFP in accordance with a particular microbe.

Another possible limitation of our pCG-VmS plasmid and its use with native promoter fusions is its potential to dilute the native transcriptional factor population due to its multicopy number, which could, in turn, affect gene expression of other promoter targets and influence reporter results. Similarly, translational fusions using multicopy plasmids such as pCG-VmS could sequester cellular translational resources and, if too much protein is being produced, result in aggregation or even artifactual results. To the latter point, GFP fusions produced in excess are well known to affect protein behavior in both prokaryotic and eukaryotic systems (50–52). These considerations indeed present a double-edged sword when using fluorophores: on the one hand, they serve as powerful imaging tools for tracking expression and localization, while on the other hand, they can produce false results and/or impose a fitness cost *in vitro* or *in vivo* (e.g., see Movie S1). Thus, there is an important balance to strike when considering the strength of the promoter used, ribosome binding site selection, and plasmid copy number.

One remedy for these aforementioned issues would be to integrate the reporter into the bacterial chromosome as a single copy. A good system to accomplish this would be the mini-CTX1 system that harbors gentamicin resistance. This system has the ability to integrate as a single-copy into the *P. aeruginosa* genome at the *attB* phage site (53) and could therefore be utilized to make single-copy reporter constructs in *P. aeruginosa*. Indeed, the *attB* site has been previously utilized to constitutively express fluorophores from the P_A1/04/03_ (strong and constitutively active promoter), as well as from native promoters (15). During the construction of pCG-VmS, a mini-CTX1 version was created as an intermediate plasmid. The feasibility of utilizing mini-CTX1-Gm-P_A1/04/03_-G-VmS in making dual-fluorophore reporter constructs *in cis* is still under investigation by our lab and can come with its own problems. For example, one must sacrifice fluorescent signal level (i.e., one copy versus up to 30 copies difference for medium copy number plasmids) to avoid deleterious effects from overexpression. Such reduction in signal becomes especially important during longitudinal microscopy (due to inevitable photobleaching) and background fluorescence from tissues and whole animals. One remedy might be to utilize a far-red fluorophore such as mKate2, or near-infrared fluorophores such as the “iRFP” or “Wi-Phy” versions derived from phytochromes and related photoreceptors (54–56). Indeed, so-called phytochrome-based fluorophore (PBF) probes have already been shown to work in *P. aeruginosa* by our group (34). However, one must proceed with caution, as PBFs utilize heme catabolites as cofactors that can affect bacterial metabolism and therefore might come with a significant fitness cost while using them during cell-based or whole-animal infection models (34).

After construction of pCG-VmS replicating plasmid, we then exploited it to verify the quiescent population of *P. aeruginosa* which had previously been reported to exist in infected corneal cells, an observation that had been observed but not verified quantitatively (12, 13). The study of these distinct populations, especially the T3SS-off bacteria, has been limited by the availability of tools to visualize both bacterial populations concurrently during infection with single-cell accuracy and, by extension, the ability to precisely quantify such heterogeneous populations. In the future, the use of this new tool will allow for further investigation of the underlying mechanisms driving these two distinct populations in their natively expressed states (i.e., in WT *P. aeruginosa*), as well as possible further dissection of the regulatory mechanisms that control T3SS activation or repression during intracellular infection. Moreover, such studies could reveal how the natural spatiotemporal T3SS induction in *P. aeruginosa* during both cellular and vertebrate infections might differ from the more unnatural *in vitro* induction by addition of EGTA or the use of T3SS pathway mutants—both strategies that have been commonly used to either induce or negate T3SS expression, respectively (57). Excitingly, here, we show for the first time that these T3SS-on and -off populations also exist during tissue infection of the excised murine eye.

In summary, the plasmid presented in this study, pCG-VmS, has proven to be a highly useful dual-reporter system that expands upon the growing genetic toolbox available for *P. aeruginosa* (3, 9, 58) and other gram-negative species (59, 60). The plasmid provides an easy means to clone promoters or make translational fusions to two fluorophores that are balanced for their optical properties (e.g., photobleaching susceptibility). The experiments performed have demonstrated its practicality in a wide variety of applications, including as an *in vitro* and *in vivo* transcriptional reporter and as a translational reporter to determine protein subcellular localization. By exploiting the pCG-VmS plasmid, we have now visualized T3SS-on and the quiescent T3SS-off populations in infected cells and vertebrate soft tissue, suggesting that *P. aeruginosa* might use a bet-hedging virulence strategy *in vivo* to avoid host assaults or antibiotic treatments. It will therefore be of great future interest to discover what host signals are driving the bimodal populations. By extension of these findings, our work here clearly shows a potential for exploiting pCG-VmS to separate niche populations of bacteria during *in vivo* and *in vitro* infection scenarios.

## MATERIALS AND METHODS

### Bacterial strains and growth conditions

Bacterial strains used in this study can be found in Table S1*. P. aeruginosa* and *E. coli* cultures were grown in Luria-Bertani medium (LB, Difco; Beckton Dickinson, Franklin Lakes, NJ). Liquid cultures were grown at 37°C and 230 rpm on an orbital shaker. *V. fischeri* cultures were grown in LB salt (LBS; 1% tryptone, 0.5% yeast extract, 2% sodium chloride, and 50 mM Tris [pH 7.5]) or Tris-minimal media (61) (TMM, 100 mM Tris pH 7.5, 300 mM NaCl, 50 mM MgSO_4_, 0.33 mM KH_2_PO_4_, 10 μM ferrous ammonium sulfate, 0.1% [wt/vol] NH_4_Cl, 10 mM N-acetylglucosamine, 10 mM KCl, and 10 mM CaCl_2_) at 28°C. Culture density was monitored using a Genesys 150 UV-Vis spectrophotometer (Thermo Fisher Scientific, Waltham, MA) at a wavelength of 600 nm. Solid medium was solidified with 1.5% agar (bacteriological; VWR, Solon, OH). Super optimal broth with catabolite repression (SOC) was prepared with 20 g/L tryptone, 5 g/L yeast extract, 0.5 g/L NaCl, 10 mM MgCl_2_, 10 mM MgSO_4_, 2.5 mM KCl, and 10 mM glucose. Antibiotic concentrations for *E. coli* were as follows: 15 μg/mL gentamicin (Gm) and 15 μg/mL chloramphenicol (Cm). For *P. aeruginosa*, Gm was used at 30 μg/mL. For *V. fischeri*, Cm was used at a concentration of 5 μg/mL.

### Fluorescence reporter assays

For *P. aeruginosa* fluorescence reporter assays, experiments were carried out as previously described (15). Briefly, *P. aeruginosa* MPAO1 cultures carrying the indicated plasmids were grown overnight and subcultured 1:100 into fresh LB medium. Cultures were then grown at 37°C and 230 rpm for 2 h before addition of compounds. Cultures were grown for an additional 3 h before being resuspended in PBS, and fluorescence readings were taken using a BioTek Synergy H1 multimode plate reader (BioTek, Winooski, VT). Green (excitation 485/20 nm, emission 528/20 nm, using a dichroic mirror 510 nm) and red (excitation 575/15 nm, emission 635/32 nm, using a dichroic mirror 595 nm) filter cubes were used for fluorescence readings. For *V. fischeri* reporter constructs, experiments were performed as in reference 35 with minor modifications. Briefly, *V. fischeri* strains were inoculated from freezer stocks into TMM media and grown at 28°C for several hours. Strains were then subcultured 1:100 into fresh TMM supplemented ± 500 µM L-cystine (HCl was utilized as a solvent control) and grown overnight. Cultures were pelleted and resuspended in PBS before fluorescence readings were performed according to the *P. aeruginosa* protocol described above.

### *P. aeruginosa* growth assays

Growth assays were performed as described previously (15). Briefly, 1 mL cultures were washed once in fresh LB medium. Cultures were then normalized to an OD_600_ of 0.05 in fresh media. Two hundred microliters of cultures was then aliquoted in triplicate into black-walled 96-well plates (655096; Greiner Bio-One, Stonehouse, UK). Growth and fluorescence were then monitored over time using a BioTek Synergy H1 multimode plate reader (BioTek, Winooski, VT) at 37°C with 567 cycles per minute (cpm) linear shaking.

### Live cell fluorescence microscopy for protein subcellular localization

Microscopy was performed as described previously (15). Briefly, an overnight culture of *P. aeruginosa* MPAO1 containing the pCG-VmS-derived reporter plasmid was subcultured 1:100 into fresh medium and grown for 2 h at 37°C and 230 rpm. The culture was diluted 1:2 in fresh LB medium, and 1–2 μL of cells were then transferred to a 1.5% agarose LB (LBA) pad on a cleaned slide. LBA pads were made by pipetting 50 μL of 1.5% agarose LB into a 125 μL Gene Frame (AB0578; Thermo Scientific) and placing a clean slide on top until the media solidified. The pad was then sealed with a No. 1.5 coverslip before imaging.

### Construction of pCG-VmS

To construct pCG-VmS, mini-CTX1-Gm-mCherry was digested with NcoI and KpnI to remove the mCherry cassette. A linear DNA fragment synthesized by Integrated DNA Technologies (IDT) containing the mScarlet-I coding sequence was PCR-amplified with primer pairs NcoI-mScarlet-I-F1/KpnI-mScarlet-I-R1 and NcoI-mScarlet-I-F2/KpnI-mScarlet-I-R2 (Table S1) and ligated into the digested plasmid to create miniCTX1-Gm-mScarlet-I. To insert the _PA1/04/03_-sfGFP cassette and 150 bp of randomly selected DNA sequence to space the divergent sfGFP and mScarlet-I genes, the reverse complement of the P_A1/04/03_-sfGFP was amplified from mini-CTX1-Gm-P_A1/04/03_-sfGFP with a 3′ sequence complementary to a noncoding section of DNA from pUC18T-mini-Tn7T-Gm with primers SacI-sfGFP-revcomp-F1 and P_A1/04/03_-150bp-pUC18T-R. A random 150 bp DNA sequence from pUC18T-mini-Tn7T-Gm was amplified using primer pairs P_A1/04/03_-150bp-pUC18T-F and NotI-150bp-pUC18T-R1, followed by splicing by overlap extension with the previously mentioned PCR product. The resulting gel-extracted DNA product was reamplified with primer pairs SacI-sfGFP-revcomp-F1/NotI-150bp-pUC18T-R2 and SacI-sfGFP-revcomp-F2/NotI-150bp-pUC18T-R1 and ligated into SacI- and NotI-digested miniCTX1-Gm-mScarlet-I to create mini-CTX1-PA_1/04/03_-G-mS.

The PA_1/04/03_-sfGFP-MCS-mScarlet-I cassette was amplified using primer pairs SacI-sfGFP-revcomp-F1/KpnI-mScarlet-I-R2 and SacI-sfGFP-revcomp-F2/KpnI-mScarlet-I-R1 and inserted into SacI- and KpnI-digested pCC21 to create pP_A1/04/03_-G-VmS. The P_A1/04/03_ promoter was then swapped for the lacUV5 promoter by digesting pP_A1/04/03_-G-VmS with PfoI and SapI and inserting the SapI- and PfoI-digested P_lacUV5_ gene fragment (synthesized by IDT) to create pP_lacUV5_-G-VmS. Finally, an rrnB terminator was amplified using primer pairs rrnBTerm-Gibson-F/rrnBTerm-Gibson-R from pMMB67EH and inserted at the PfoI site between the divergent genes in the direction of the sfGFP coding sequence by Gibson assembly to yield pCG-VmS.

Transcriptional fusions were made in pCG-VmS by amplifying the upstream regulatory region with primers containing 5′ BamHI and 3′ HindIII overhangs and inserting the product BamHI- and HindIII-digested pCG-VmS. Primer pairs for P*_arqI_* (145 bp) were BamHI-P_ArqI_-F1/HindIII-P_ArqI_-R2 and BamHI-P_arqI_-F2/HindIII-P_ArqI_-R1. Primer pairs for P_exoS_ amplification from pJNE05 (62) were BamHI-P_exoS_-F1/HindIII-P_exoS_-R2 and BamHI-P_exoS_-F2/HindIII-P_exoS_-R1. Translational fusions of ArqI to mScarlet-I were made by amplifying the *arqI* upstream regulatory region (145 bp), *arqI* gene, and a C-terminal domain-breaking linkerfrom pSB109-ArqI-sfGFP using primer pairs BamHI-P_arqI_-F1/NcoI-Linker-R2 and BamHI-P_ArqI_-F1/NcoI-LinkerR1. The resulting product was ligated into BamHI- and NcoI-digested pCG-VmS to yield pCG-P*_arqI_*-ArqI-mS.

### Construction of flow cytometry control plasmids

For flow cytometry controls, pSB109-sfGFP and pSB109-mScarlet-I were constructed. The sfGFP coding sequence was amplified using primer pairs NcoI-sfGFP-F1/NdeI-sfGFP-R2 and NcoI-sfGFP-F2/NdeI-sfGFP-R1. The resulting product was then ligated into NcoI- and NdeI-digested pSB109 using a restrictionless cloning method (63). Similarly, the mScarlet-I coding sequence was amplified with primer pairs NcoI-mScarlet-I-F1/NdeI-mScarlet-I-R2 and NcoI-mScarlet-I-F2/NdeI-mScarlet-I-R1 and ligated into pSB109.

### Construction of pVSV105-CG-VmS

To construct pVSV105-CG-VmS, the native NcoI site in pVSV105 was disrupted using site-directed mutagenesis by the QuickChange Method (64) using primers pVSV105-NcoI-Poison-F and pVSV105-NcoI-Poison-R (Table S1). The resulting plasmid was digested by NotI and SphI followed by insertion of a PCR-amplified copy of P_A1/04/03_-sfGFP from pP_A1/04/03_-G-VmS using primer pairs NotI-sfGFP-F1/SphI-sfGFP-R2 and NotI-sfGFP-F2/SphI-sfGFP-R1 to yield pVSV105-CG. pVSV105-CG was digested with SacI and SpeI before insertion of a PCR-amplified copy of the RBS and mScarlet-I genes, amplified from pP_A1/04/03_-G-VmS using the primer pairs SacI-RBS-mScarlet-I-F1/SpeI-mScarlet-R2 and SacI-RBS-mScarlet-I-F2/SpeI-mScarlet-R1, to yield pVSV105-CG-VmS. Transcriptional fusions were made by insertion of upstream regulatory regions at the SphI and KpnI sites using the following primers: P_rpoQ_, SphI-P_rpoQ_-F/KpnI-P_rpoQ_-R; P_cysK_, SphI-P_cysK_-F/KpnI-P_cysK_-R (Table S1).

### Flow cytometry

Cultures were grown as described in the “Fluorescence reporter assays” section. Samples were diluted to a cell density of ~5 × 10^7^ CFU/mL (OD_600_ 0.1 = 1 × 10^8^ CFU/mL) in 2 mL PBS. Samples were read on a full-spectrum cytometer, the 5-laser Aurora (Cytek, Fremont, CA). Appropriate sfGFP and mScarlet-I single-color controls were used to unmix and gate the data. Analyses of the unmixed data were performed in FlowJo v10.9.0 (BD Life Sciences, Ashland, OR). sfGFP-positive cells were gated on for all analyses.

### Biofilm flow cell imaging

Flow cell bioreactors were prepared as previously described (65). Log-phase MPAO1 pCG-ParqI-mS cultures grown in tryptic soy broth (TSB) were diluted to 0.01 OD_600_ in 1% TSB. Diluted cultures were injected into continuous flow cell chambers, which were then inverted and incubated for 1 h at room temperature to allow for cell attachment. Following attachment, continuous flow of 1% TSB media was supplied at a rate of 10 mL/h for 96 h at room temperature. Resultant biofilms were treated with 10 mM GO in 1% TSB under 10 mL/h flow for 3 h and subsequently imaged using confocal microscopy (Nikon A1R). Three images were taken for each sample in three independent experiments. Representative z-stack images were processed in Volocity Image Analysis (Improvision, Coventry, UK).

### Squid colonization experiments

Hatchling *E. scolopes* were colonized with *V. fischeri* KV10353 by the addition of 3,000 CFU/mL to filter-sterilized artificial seawater (FSASW) for 3 h, followed by transfer to 20 mL glass scintillation vials containing FSASW. Light organ colonization was determined at 24 h post-inoculation by monitoring luminescence using a Turner TD 202/20 single-tube luminometer (Turner Instruments). Aposymbiotic control animals were maintained in FSASW without the addition of *V. fischeri* KV10353. Symbiotic animals were anesthetized in 2% ethanol in FSASW and then fixed for 12 hours in 4% paraformaldehyde in FSASW at 4°C. Fixed animals were then rinsed and stored in FSASW until imaging.

For imaging, animals were mounted in 1× marine phosphate-buffered saline (50 mM sodium phosphate, 0.45 M NaCl, pH 7.4) on concavity well slides (Electron Microscopy Sciences). Mounted animals were then imaged using a Nikon A1R laser-scanning confocal microscope with 10×/0.30 NA Plan Fluorite and 40×/1.20 NA Plan Apochromat objectives at the University of Connecticut Advanced Light Microscopy Facility. Images for sfGFP and mScarlet-I signals were acquired using 488 and 561 nm laser lines, respectively, to excite the fluorophores.

### Cell culture and time-lapse imaging

hTCEpi cells were maintained in KGM-2 media (PromoCell) (66). HeLa cells were maintained in phenol red-free DMEM (Gibco) containing 10% fetal bovine serum (FBS). One day preceding the experiments, hTCEpi cells were plated on No. 1.5 glass-bottom 24-well plates (MatTek) in KGM-2 at 75% confluence with 1.06 mM calcium to induce differentiation (66). HeLa cells were plated at 50% confluence on optical plastic 8-well chambered coverslips (Ibidi). For infection, bacterial suspensions were made in PBS from 16-hour lawns grown on TSA medium containing 100 µg/mL gentamicin at 37°C. A multiplicity of infection of 10 was calculated using an OD_540_ of 1, equivalent to 4 × 10^8^ CFU/mL. Bacteria were added directly to culture media and allowed to invade cells. The medium was replaced with medium containing amikacin (200 µg/mL) or additional polymyxin B (10 µg/mL) at 3 h post-infection to kill extracellular bacteria. Of note, this time point precedes observed fluorescence of reporters for ExoS, and this technique has been validated to observe intracellular bacteria previously (26). Beginning at 3.5 h post-infection, images were captured on a Ti2-E inverted microscope with X-Cite XYLIS XT720S Broad Spectrum LED Illumination System, equipped with an Okolab stage-top incubation chamber to maintain 37°C and 5% CO_2_, a DS-Qi2 CMOS camera, and CFI Plan Apochromat Lambda D 40× air NA 0.95 objective. Time-lapse fields were selected without observing fluorescence channels to limit bias in field selection. Images were captured with intervals of 5 or 10 min.

### *P. aeruginosa ex vivo* mouse cornea infection

*Ex vivo* corneal infections were performed as described previously with minor modifications (67). Briefly, 8- to 12-week-old C57/BL6 mice were sacrificed by cervical dislocation following CO_2_ asphyxiation. Whole eyes were carefully enucleated with curved forceps. A horizontal “scratch” with a 26 G needle was then performed on the cornea, followed by three light blots with a Kimwipe (Kimberly-Clark). The eyes were placed into a 96-well tissue culture dish and rinsed three times by dipping into in phosphate-buffered saline (PBS), followed by a 1-hour room-temperature incubation with 100 mM EGTA to disrupt epithelial cell-cell junctions, allowing bacterial translocation (67, 68). After EGTA treatment, eyes were again washed three times in PBS and then placed in serum-free phenol, red-free DMEM (Gibco), containing 4 × 10^7^ CFU of either wild-type or Δ*exsA* PAO1f pCG-P*_exoS_*-mS for 6 h at 30°C and 5% CO_2_. Uninfected eyes were incubated in DMEM for the same amount of time.

To stain corneal epithelial tissue post-infection, eyes were removed from DMEM and placed in a 1:2,000 solution (final concentration of 0.5 µg/mL) of AlexaFluor 647-labeled wheat germ agglutinin (Invitrogen) in PBS for 5 min, followed by three PBS washes and a 1-hour fixation in a 1.3% solution of diluted paraformaldehyde (Thermo Scientific) in PBS. Eyes were then mounted (cornea face up) on a plastic coverslip, submerged in PBS, and imaged on a Nikon Ti-E spinning disk confocal microscope equipped with an Apochromat 60× water-dipping objective lens (N.A. 1.0), a Lumencor CELESTA Light Engine, a Crest X-Light V2, and a Photometrics Prime CMOS Camera.

## Data Availability

Further information and requests for resources and reagents should be directed to and will be fulfilled by the lead contact, Andrew T. Ulijasz (aulijasz@uab.edu). Unique reagents are available through an MTA.

## SUPPLEMENTAL MATERIAL

Supplemental figures aem.00490-26-s0001.pdf Fig. S1 and S2; legends for Movies S1 to S5.

Table S1 aem.00490-26-s0002.xlsx Primers, plasmids, and strains.

Movie S1 aem.00490-26-s0003.mp4 Optimization of a constitutive GFP promoter.

Movie S2 aem.00490-26-s0004.mp4 Comparison of pCG-P*_exoS_*-mS to a previously characterized reporter for T3SS activity.

Movie S3 aem.00490-26-s0005.mp4 Amikacin and polymyxin B eliminate extracellular bacteria.

Movie S4 aem.00490-26-s0006.mp4 Z-stack of *ex vivo* scratched C57/BL6 mouse eye infection with wild-type PAO1f pCG-P*_exoS_*-mS.

Movie S5 aem.00490-26-s0007.mp4 Z-stack of *ex vivo* scratched C57/BL6 mouse eye infection with Δ*exsA* (T3SS-null) PAO1f pCG-P*_exoS_*-mS.

ASM does not own the copyrights to Supplemental Material that may be linked to, or accessed through, an article. The authors have granted ASM a non-exclusive, world-wide license to publish the Supplemental Material files. Please contact the corresponding author directly for reuse.
