# Supplementary material for: A versatile dual-color bacterial reporter system highlights two distinct Pseudomonas aeruginosa type 3 secretion system intracellular populations
Source: Appl Environ Microbiol. 2026 Jun 3;92(7):e00490-26. doi: 10.1128/aem.00490-26 (PMC13390426; doi:10.1128/aem.00490-26)
Supplement: Supplemental figures — Fig. S1 and S2; legends for Movies S1 to S5. [file aem.00490-26-s0001.pdf]

Supplemental Figure 1

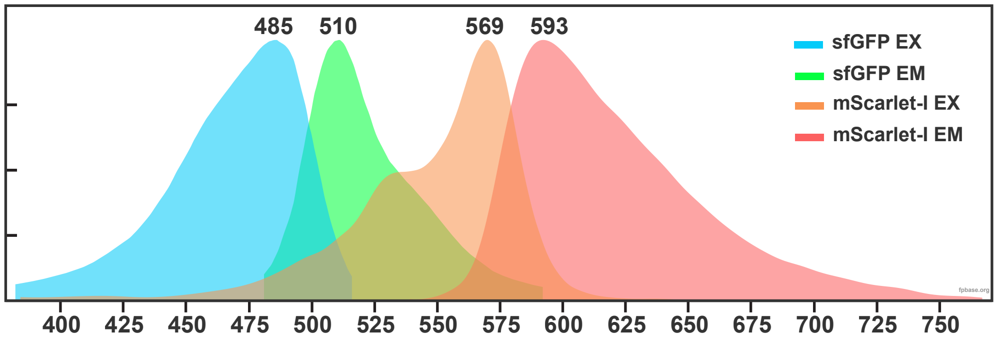

Supplemental Figure 2

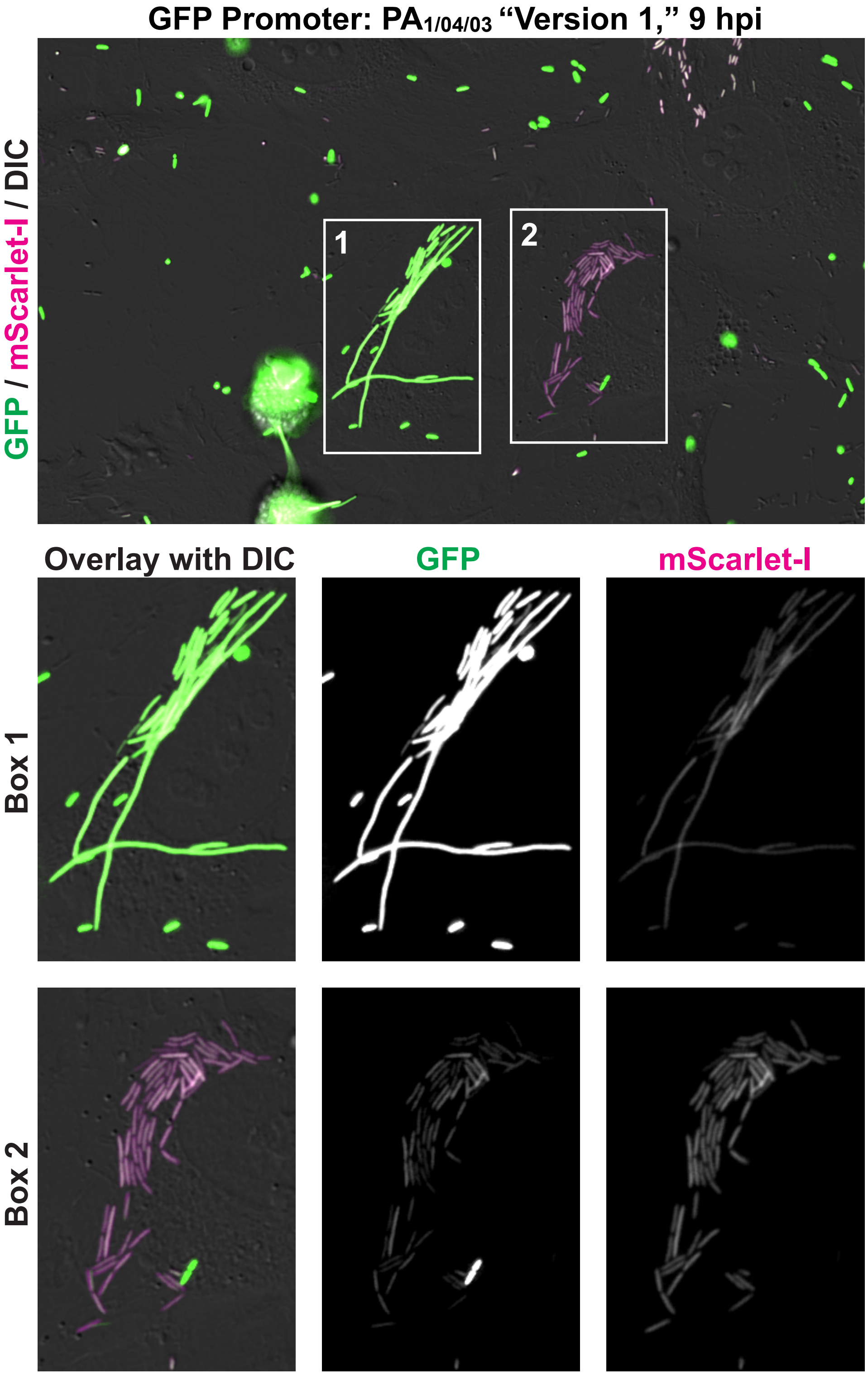

A versatile dual-color reporter reveals a bimodal Type-III Secretion System distribution in Pseudomonas aeruginosa during infection

Christopher J. Corcoran[1,2], David G. Glanville[3], Zachary J. Resko[1], Erin K. Cassin[4], Andrew D. Marten[1], Derrick L. Kamp[5], Karen L. Visick[1], Spencer V. Nyholm[5], Boo Shan Tseng[4], Abby R. Kroken[1*], Andrew T. Ulijasz[3,6*]

Supplemental Figure Legends

Supplemental Figure 1. Excitation (Ex) and emission (Em) spectra of sfGFP and mScarlet-I. The figure was built from spectra downloadeds from FPbase.org.

Supplemental Figure 2. Still of Supplemental Movie 1. PAO1F Δ*exoSTY* using promoter $PA_{1/04/03}$ to drive mScarlet-I expression in infected HeLa cells.

Supplemental Movie 1. Optimization of a constitutive GFP promotor. PAO1FΔ*exoSTY* was transformed with either $PA_{1/04/03}$ (labeled “version 1”) or pCG-VmS (labeled “version 2”), each encoding the promotor for ExoS upstream of mScarlet-I (pCG-P_{exoS}-mS). Bacteria were incubated with HeLa cells for 3 hours to allow internalization. Planktonic bacteria were removed and media replaced with amikacin-containing media. Time-lapse images were captured every 10 minutes to track intracellular bacterial replication fluorophore expression. Labeling of hemagglutinin is shown in purple. Bacteria that appear yellow are expressing mScarlet-I indicating *exoS* promoter (P_{exoS}) activation. Notice the heterogeneity in *exoS* expression. sfGFP and mScarlet-I signal were scaled identically in these visualizations.

Supplemental Movie 2. Comparison of pCG-P_{exoS}-mS to a previously characterized reporter for T3SS activity. PAO1F was transformed with either PJNE05 (encodes the promotor for ExoS upstream of GFP) or pCG-P_{exoS}-mS. Bacteria were incubated with hTCEpi cells for 3 hours to allow internalization. Planktonic bacteria were removed and media replaced with amikacin-containing media. Time-lapse images were captured every 5 minutes to compare the two fluorescent reporters in intracellular populations.

Supplemental Movie 3. Amikacin and Polymyxin B eliminate extracellular bacteria. PAO1F transformed with pCG-P*exoS*-mS was incubated with hTCEpi cells for 3 hours to allow internalization. Planktonic bacteria were removed and media replaced with amikacin and polymyxin B-containing media, which causes extracellular bacteria to lyse and diminishes their fluorescence within 1-2 hours. Time-lapse images were captured every 5 minutes.

Supplemental Movie 4. Z-stack of *ex vivo* scratched C57/BL6 mouse eye infection with wild-type PAO1f pCG-P*exoS*-mS. Labeling of hemagglutinin is shown in purple. Bacteria that appear yellow are expressing mScarlet-I indicating *exoS* promoter (P*exoS*) activation. Notice the heterogeneity in *exoS* expression. sfGFP and mScarlet-I signal were scaled identically in these visualizations.

Supplemental Movie 5. Z-stack of *ex vivo* scratched C57/BL6 mouse eye infection with *ΔexsA* (T3SS-null) PAO1f pCG-P*exoS*-mS. Labeling of hemagglutinin is shown in purple. Bacteria that appear yellow are expressing mScarlet-I indicating *exoS* promoter (P*exoS*) activation. Notice the heterogeneity in *exoS* expression. sfGFP and mScarlet-I signal were scaled identically in these visualizations.
